# Supplementary material for: Genetic epidemiology of dengue viruses in phase III trials of the CYD tetravalent dengue vaccine and implications for efficacy
Source: eLife. 2017 Sep 5;6:e24196. doi: 10.7554/eLife.24196 (PMC5584992; doi:10.7554/eLife.24196)
Supplement: Supplementary file 1 . — (b) Mean percent identity between E gene amino acid sequences of the relevant serotype-specific CYD-TDV vaccine strain and virus populations sampled in CYD14/15. (c) Number of E gene sequences per genotype per country in CYD-TDV trials versus publicly available sequences on GenBank. I. CYD14, II. CYD15. (1d) Variation in the number of cases, imputed versus observed. [file elife-24196-supp1.docx]

**Supplementary File 1a – Sequencing success rates in samples from virologically confirmed dengue cases in vaccine and control groups from the CYD-TDV trials.**

|  | **Total** | **Vaccine** | **Control** |
| --- | --- | --- | --- |
| **CYD14** | | | |
| Number of VCD samples (total sequencing attempts) | 432 | 211 | 221 |
| Sequences generated | 324 | 152 | 172 |
| Sequencing success rate | 75.0% | 72.0% | 77.8% |
| Sequencing failure rate | 25.0% | 28.0% | 22.2% |
| **CYD15** | | | |
| Number of VCD samples (total sequencing attempts) | 513 | 210 | 303 |
| Sequences generated | 340 | 136 | 204 |
| Sequencing success rate | 66.3% | 64.8% | 67.3% |
| Sequencing failure rate | 33.7% | 35.2% | 32.7% |
| **CYD14+CYD15** | | | |
| Number of VCD samples (total sequencing attempts) | 945 | 421 | 524 |
| Sequences generated | 664 | 288 | 376 |
| Sequencing success rate | 70.3% | 68.4% | 71.8% |
| Sequencing failure rate | 29.7% | 31.6% | 28.2% |

**Supplementary File 1b – Mean percent identity between E gene amino acid sequences of the relevant serotype-specific CYD-TDV vaccine strain and virus populations sampled in CYD14/15.**

|  | | **Amino acid identity across the E gene relative to relative to the CYD vaccine sequence** | | |
| --- | --- | --- | --- | --- |
|  |  |  |  |  |
|  | | **Average (%)** | **Minimum (%)** | **Maximum (%)** |
| Serotype 1 |  | | | |
|  | Genotype I CYD14 | 97.81 | 97.17 | 98.18 |
|  | Genotype IV CYD14 | 97.09 | 96.16 | 97.37 |
|  | Genotype V CYD15 | 97.02 | 96.16 | 97.37 |
| Serotype 2 |  | | | |
|  | American/Asian CYD15 | 97.93 | 97.17 | 98.18 |
|  | Asian 1 CYD14 | 97.96 | 96.16 | 98.38 |
|  | Cosmopolitan CYD14 | 97.46 | 95.96 | 98.38 |
| Serotype 3 |  | | | |
|  | Genotype I CYD14 | 97.99 | 97.16 | 98.17 |
|  | Genotype II CYD14 | 98.28 | 98.17 | 98.38 |
|  | Genotype III CYD14 | 97.57 | 97.57 | 97.57 |
|  | Genotype III CYD15 | 97.83 | 97.16 | 97.97 |
| Serotype 4 |  | | | |
|  | Genotype I CYD14 | 97.68 | 96.77 | 98.79 |
|  | Genotype II CYD14 | 99.10 | 98.59 | 99.39 |
|  | Genotype II CYD15 | 97.82 | 96.15 | 97.98 |

**Supplementary File 1c – Number of E gene sequences per genotype in CYD-TDV trials versus publicly available sequences on GenBank. I. CYD14, II. CYD15.**

**I.**

|  | | **Vietnam** | | **Thailand** | | **Malaysia** | | **Indonesia** | | **The Philippines** | |
| --- | --- | --- | --- | --- | --- | --- | --- | --- | --- | --- | --- |
| **CYD14** | | **CYD14 sequences** | **Number of sequences on GenBank originating from 2010-2014 (proportion)** | **CYD14 sequences** | **Number of sequences on GenBank originating from 2010-2014 (proportion)** | **CYD14 sequences** | **Number of sequences on GenBank originating from 2010-2014 (proportion)** | **CYD14 sequences** | **Number of sequences on GenBank originating from 2010-2014 (proportion)** | **CYD14 sequences** | **Number of sequences on GenBank originating from 2010-2014 (proportion)** |
| **Serotype 1** |  | | | | | | | | | | |
|  | **Genotype I** | 7 | 91 (1) | 15 | 11 (1) | 2 | 41 (0.71) | 9 | 41 (0.55) |  |  |
|  | **Genotype II** |  |  |  |  |  |  |  | 1 (0.01) |  |  |
|  | **Genotype III** |  |  |  |  |  |  |  |  |  |  |
|  | **Genotype IV** |  |  |  |  |  | 2 (0.03) | 3 | 32 (0.43) | 88 | 9 (1) |
|  | **Genotype V** |  |  |  |  |  | 15 (0.26) |  |  |  |  |
| **Serotype 2** |  | | | | | | | | | | |
|  | **Asian/American** |  |  |  |  |  |  |  |  |  |  |
|  | **Asian I** | 15 | 85 (0.97) | 24 | 13 (0.81) |  | 3 (0.04) |  |  |  |  |
|  | **Cosmopolitan** |  | 3 (0.03) |  | 3 (0.19) | 2 | 80 (0.96) | 9 | 72 (1) | 38 | 13 (1) |
|  | **Asian II** |  |  |  |  |  |  |  |  |  |  |
|  | **American** |  |  |  |  |  |  |  |  |  |  |
| **Serotype 3** |  | | | | | | | | | | |
|  | **Genotype I** |  |  |  |  |  | 1 (0.25) | 5 | 18 (1) | 18 | 4 (0.8) |
|  | **Genotype II** | 4 | 5 (0.63) |  | 3 (0.43) |  |  |  |  |  | 1 (0.2) |
|  | **Genotype III** |  | 3 (0.37) | 11 | 4 (0.57) |  | 3 (0.75) |  |  |  |  |
|  | **Genotype IV** |  |  |  |  |  |  |  |  |  |  |
|  | **Genotype V** |  |  |  |  |  |  |  |  |  |  |
| **Serotype 4** |  | | | | | | | | | | |
|  | **Genotype I** | 28 | 46 (1) | 2 | 2 (1) |  |  |  |  | 3 |  |
|  | **Genotype II** |  |  |  |  | 5 | 1 (1) | 1 | 18 (1) | 25 | 1 (1) |
|  | **Genotype III** |  |  |  |  |  |  |  |  |  |  |
|  | **Genotype IV** |  |  |  |  |  |  |  |  |  |  |
|  | **Genotype V** |  |  |  |  |  |  |  |  |  |  |

**II.**

|  | | **Mexico** | | **Brazil** | | **Honduras** | | **Puerto Rico** | | **Colombia** | |
| --- | --- | --- | --- | --- | --- | --- | --- | --- | --- | --- | --- |
| **CYD15** | | **CYD15 sequences** | **Number of sequences on GenBank originating from 2010-2014 (proportion)** | **CYD15 sequences** | **Number of sequences on GenBank originating from 2010-2014 (proportion)** | **CYD15 sequences** | **Number of sequences on GenBank originating from 2010-2014 (proportion)** | **CYD15 sequences** | **Number of sequences on GenBank originating from 2010-2014 (proportion)** | **CYD15 sequences** | **Number of sequences on GenBank originating from 2010-2014 (proportion)** |
| **Serotype 1** |  | | | | | | | | | | |
|  | **Genotype I** |  |  |  |  |  |  |  |  |  |  |
|  | **Genotype II** |  |  |  |  |  |  |  |  |  |  |
|  | **Genotype III** |  |  |  |  |  |  |  |  |  |  |
|  | **Genotype IV** |  |  |  |  |  |  |  |  |  |  |
|  | **Genotype V** | 32 | 40 (1) | 15 | 27 (1) | 3 |  | 14 | 36 (1) | 65 |  |
| **Serotype 2** |  | | | | | | | | | | |
|  | **Asian/American** | 42 | 9 (1) | 1 | 6 (1) | 19 |  |  | 5 (1) | 34 |  |
|  | **Asian I** |  |  |  |  |  |  |  |  |  |  |
|  | **Cosmopolitan** |  |  |  |  |  |  |  |  |  |  |
|  | **Asian II** |  |  |  |  |  |  |  |  |  |  |
|  | **American** |  |  |  |  |  |  |  |  |  |  |
| **Serotype 3** |  | | | | | | | | | | |
|  | **Genotype I** |  |  |  |  |  |  |  |  |  |  |
|  | **Genotype II** |  |  |  |  |  |  |  |  |  |  |
|  | **Genotype III** |  |  |  |  | 19 |  |  |  | 50 |  |
|  | **Genotype IV** |  |  |  |  |  |  |  |  |  |  |
|  | **Genotype V** |  |  |  |  |  |  |  |  |  |  |
| **Serotype 4** |  | | | | | | | | | | |
|  | **Genotype I** |  |  |  | 1 (0.01) |  |  |  |  |  |  |
|  | **Genotype II** |  |  | 34 | 130 (0.99) |  |  | 1 | 18 (1) | 3 |  |
|  | **Genotype III** |  |  |  |  |  |  |  |  |  |  |
|  | **Genotype IV** |  |  |  |  |  |  |  |  |  |  |
|  | **Genotype V** |  |  |  |  |  |  |  |  |  |  |

**Supplementary File 1d. Variation in the number of cases, imputed versus observed.**

|  | | **CYD Dengue Vaccine Group** | | **Control Group** | |
| --- | --- | --- | --- | --- | --- |
|  |  |  |  |  |  |
|  | | **Cases observed** | **Cases imputed** | **Cases observed** | **Cases imputed** |
| Serotype 1 |  | | | | |
|  | Genotype I CYD14 | 15 | 25-35 | 18 | 32-36 |
|  | Genotype IV CYD14 | 40 | 84-91 | 51 | 90-94 |
|  | Genotype V CYD15 | 53 | 99 | 76 | 109 |
| Serotype 2 |  | | | | |
|  | American/Asian CYD15 | 48 | 84 | 50 | 84 |
|  | Asian I CYD14 | 28 | 45 | 14 | 28 |
|  | Cosmopolitan CYD14 | 28 | 52 | 21 | 46 |
| Serotype 3 |  | | | | |
|  | Genotype I CYD14 | 9 | 21 | 14 | 25 |
|  | Genotype II CYD14 | 0 | 2 | 4 | 7 |
|  | Genotype III CYD14 | 4 | 7 | 7 | 11 |
|  | Genotype III CYD15 | 23 | 55 | 47 | 106 |
|  | Genotype III CYD14+CYD15 | 27 | 62 | 54 | 117 |
| Serotype 4 |  | | | | |
|  | Genotype I CYD14 | 19 | 26-30 | 18 | 30-38 |
|  | Genotype II CYD14 | 8 | 10-14 | 24 | 34-42 |
|  | Genotype II CYD15 | 11 | 32 | 31 | 83 |
|  | Genotype II CYD14+CYD15 | 19 | 42-46 | 55 | 117-125 |
