# Supplementary material for: Genetic epidemiology of dengue viruses in phase III trials of the CYD tetravalent dengue vaccine and implications for efficacy
Source: eLife. 2017 Sep 5;6:e24196. doi: 10.7554/eLife.24196 (PMC5584992; doi:10.7554/eLife.24196)
Supplement: Supplementary file 2 . [file elife-24196-supp2.docx]

**Supplementary File 2. Observed and imputed efficacy of CYD-TDV for subjects less than 9 years of age who received** ≥**1 injection (intention to treat) by serotype and genotype.**

|  | | **CYD Dengue Vaccine Group** | | | **Control Group** | | | **Vaccine Efficacy**  **Observed** | | **Vaccine Efficacy**  **With imputation** | |
| --- | --- | --- | --- | --- | --- | --- | --- | --- | --- | --- | --- |
|  | | **Cases** | **Person-years at risk** | **Density incidence (95% CI)** | **Cases** | **Person-years at risk** | **Density incidence (95% CI)** | **%** | **(95% CI)** | **%** | **(95% CI)** |
| Serotype 1 |  | | | | | | | 51.8 | (25.8; 68.7) | 46.5 | (26.5; 61.0) |
|  | Genotype I CYD14 ^CYD^ | 9 | 7060 | 0.1 (0.1; 0.2) | 10 | 3490 | 0.3 (0.1; 0.5) | 55.6 | (-10.1; 82.4) | 45.9 | (-7.7; 72.8) |
|  | Genotype IV CYD14 | 32 | 7060 | 0.5 (0.3; 0.6) | 32 | 3490 | 0.9 (0.6; 1.3) | 50.6 | (19.2; 69.8) | 46.9 | (23.2; 63.2) |
|  | p-value * |  |  |  |  |  |  | 0.840 |  | 0.7585 |  |
| Serotype 2 |  | | | | | | | 11.8 | (-51.1; 47.2) | 33.6 | (3.0; 54.2) |
|  | Asian 1 CYD14 ^CYD^ | 16 | 7079 | 0.2 (0.1; 0.4) | 5 | 3526 | 0.1 (0.0; 0.3) | -59.6 | (-388; 37.4) | 0.1 | (-107; 49.1) |
|  | Cosmopolitan CYD14 | 23 | 7079 | 0.3 (0.2; 0.5) | 17 | 3526 | 0.5 (0.3; 0.8) | 32.8 | (-27.7; 63.9) | 44.7 | (12.9; 64.8) |
|  | p-value * |  |  |  |  |  |  | 0.149 |  | 0.161 |  |
| Serotype 3 |  | | | | | | | 71.6 | (33.6; 88.6) | 62.1 | (31.5; 79.4) |
|  | Genotype I CYD14 | 5 | 7120 | <0.1 (0.0; 0.2) | 8 | 3548 | 0.2 (0.1; 0.4) | 68.8 | (6.5; 90.6) | 56.3 | (10.3; 79.0) |
|  | Genotype II CYD14 ^CYD^ | 0 | 7120 | 0.0 (0.0; 0.1) | 1 | 3548 | <0.1 (0.0; 0.2) | 100.0 | (-191; 100.0) | 100.0 | (19.3; 100.0) |
|  | Genotype III CYD14 | 3 | 7120 | <0.1 (0.0; 0.1) | 5 | 3548 | 0.1 (0.0; 0.3) | 70.3 | (-21.1; 93.9) | 64.6 | (-11.0; 89.5) |
|  | p-value * |  |  |  |  |  |  | 1.000 |  | 0.4318 |  |
| Serotype 4 |  | | | | | | | 34.5 | (-27.4; 65.7) | 51.7 | (20.1; 70.9) |
|  | Genotype I CYD14 | 16 | 7110 | 0.2 (0.1; 0.4) | 6 | 3547 | 0.2 (0.1; 0.4) | -33.2 | (-271; 45.2) | 16.9 | (-69.4; 59.3) |
|  | Genotype II CYD14 ^CYD^ | 5 | 7110 | <0.1 (0.0; 0.2) | 10 | 3547 | 0.3 (0.1; 0.5) | 75.1 | (29.9; 92.2) | 76.8 | (45.5; 90.1) |
|  | p-value * |  |  |  |  |  |  | 0.018 |  | 0.0246 |  |

Cases: number of subjects with at least one sequenced symptomatic virologically-confirmed dengue episode during the active phase of follow-up.

Density incidence: data indicate cases per 100 person-years at risk.

* The p-value was obtained by testing the heterogeneity of genotype distribution between groups (within each serotype) using a Chi^2^ (or Fisher’s exact test).

^CYD^ Genotype of the serotype-specific CYD-TDV vaccine component.
