# Supplementary material for: Genetic epidemiology of dengue viruses in phase III trials of the CYD tetravalent dengue vaccine and implications for efficacy
Source: eLife. 2017 Sep 5;6:e24196. doi: 10.7554/eLife.24196 (PMC5584992; doi:10.7554/eLife.24196)
Supplement: Supplementary file 3. [file elife-24196-supp3.docx]

**Supplementary File 3. Phred scores indicating sequence quality for all CYD14/15 DENV prM/E sequences.**

| **Sequence ID code** | **%Q20** | **%Q30** | **%Q40** |
| --- | --- | --- | --- |
| DV1/BRAZIL/122/2013 | 99.7% | 99.3% | 99.0% |
| DV1/BRAZIL/123/2013 | 99.8% | 99.3% | 98.8% |
| DV1/BRAZIL/124/2012 | 99.9% | 99.6% | 99.4% |
| DV1/BRAZIL/125/2012 | 99.9% | 99.7% | 99.4% |
| DV1/BRAZIL/145/2013 | 98.5% | 95.4% | 92.0% |
| DV1/BRAZIL/146/2013 | 99.8% | 99.3% | 97.1% |
| DV1/BRAZIL/147/2012 | 99.9% | 99.4% | 99.1% |
| DV1/BRAZIL/148/2013 | 95.3% | 94.5% | 92.8% |
| DV1/BRAZIL/149/2013 | 99.8% | 99.7% | 99.5% |
| DV1/BRAZIL/150/2012 | 99.7% | 99.4% | 99.2% |
| DV1/BRAZIL/151/2012 | 99.7% | 99.3% | 98.8% |
| DV1/BRAZIL/152/2012 | 99.7% | 99.2% | 98.9% |
| DV1/BRAZIL/349/2012 | 99.7% | 99.4% | 99.0% |
| DV1/BRAZIL/350/2012 | 99.8% | 99.4% | 99.2% |
| DV1/BRAZIL/351/2013 | 99.7% | 99.4% | 99.0% |
| DV1/COLOMBIA/126/2012 | 99.8% | 99.3% | 99.0% |
| DV1/COLOMBIA/127/2013 | 99.8% | 99.5% | 98.9% |
| DV1/COLOMBIA/128/2012 | 99.9% | 99.4% | 99.2% |
| DV1/COLOMBIA/129/2013 | 99.6% | 99.2% | 99.0% |
| DV1/COLOMBIA/130/2013 | 99.7% | 99.5% | 99.1% |
| DV1/COLOMBIA/131/2013 | 98.7% | 97.7% | 97.0% |
| DV1/COLOMBIA/132/2013 | 99.7% | 98.4% | 97.6% |
| DV1/COLOMBIA/133/2013 | 99.8% | 99.6% | 99.5% |
| DV1/COLOMBIA/134/2013 | 99.7% | 99.4% | 99.3% |
| DV1/COLOMBIA/135/2013 | 99.6% | 99.4% | 99.0% |
| DV1/COLOMBIA/136/2013 | 99.7% | 99.4% | 99.0% |
| DV1/COLOMBIA/137/2013 | 99.3% | 99.0% | 98.8% |
| DV1/COLOMBIA/138/2013 | 99.8% | 99.6% | 99.4% |
| DV1/COLOMBIA/153/2013 | 99.9% | 99.7% | 99.7% |
| DV1/COLOMBIA/154/2013 | 99.7% | 99.5% | 99.0% |
| DV1/COLOMBIA/155/2013 | 100.0% | 99.3% | 98.8% |
| DV1/COLOMBIA/156/2013 | 98.9% | 98.2% | 98.0% |
| DV1/COLOMBIA/157/2012 | 99.7% | 99.5% | 99.2% |
| DV1/COLOMBIA/158/2013 | 99.8% | 99.5% | 99.4% |
| DV1/COLOMBIA/332/2013 | 89.6% | 82.1% | 79.5% |
| DV1/COLOMBIA/333/2013 | 96.9% | 95.2% | 94.0% |
| DV1/COLOMBIA/336/2013 | 98.7% | 95.7% | 93.2% |
| DV1/COLOMBIA/337/2012 | 98.1% | 96.2% | 95.1% |
| DV1/COLOMBIA/338/2013 | 99.8% | 99.2% | 98.5% |
| DV1/COLOMBIA/339/2012 | 96.3% | 91.8% | 89.6% |
| DV1/COLOMBIA/340/2013 | 99.4% | 99.1% | 98.5% |
| DV1/COLOMBIA/341/2013 | 99.3% | 99.0% | 98.5% |
| DV1/COLOMBIA/342/2013 | 99.8% | 99.6% | 99.4% |
| DV1/COLOMBIA/343/2012 | 99.3% | 97.9% | 96.3% |
| DV1/COLOMBIA/344/2013 | 99.6% | 99.1% | 98.8% |
| DV1/COLOMBIA/345/2013 | 99.0% | 97.6% | 97.1% |
| DV1/COLOMBIA/346/2013 | 99.7% | 99.1% | 98.4% |
| DV1/COLOMBIA/347/2012 | 99.8% | 99.4% | 99.0% |
| DV1/COLOMBIA/348/2013 | 99.5% | 99.2% | 98.7% |
| DV1/COLOMBIA/353/2013 | 99.7% | 99.3% | 99.2% |
| DV1/COLOMBIA/356/2013 | 99.7% | 99.4% | 99.0% |
| DV1/COLOMBIA/358/2013 | 96.6% | 95.8% | 95.5% |
| DV1/COLOMBIA/360/2013 | 99.8% | 99.1% | 97.1% |
| DV1/COLOMBIA/502/2012 | 99.7% | 99.4% | 99.0% |
| DV1/COLOMBIA/503/2013 | 99.4% | 99.1% | 98.6% |
| DV1/COLOMBIA/504/2013 | 99.6% | 98.8% | 98.4% |
| DV1/COLOMBIA/505/2012 | 99.7% | 99.5% | 98.9% |
| DV1/COLOMBIA/506/2013 | 99.9% | 99.6% | 99.0% |
| DV1/COLOMBIA/507/2012 | 99.7% | 99.5% | 98.6% |
| DV1/COLOMBIA/508/2013 | 99.8% | 99.2% | 98.4% |
| DV1/COLOMBIA/509/2013 | 99.8% | 99.3% | 98.9% |
| DV1/COLOMBIA/510/2013 | 98.3% | 96.8% | 96.1% |
| DV1/COLOMBIA/511/2013 | 99.6% | 99.3% | 98.9% |
| DV1/COLOMBIA/512/2013 | 99.6% | 99.1% | 98.5% |
| DV1/COLOMBIA/513/2013 | 99.8% | 99.6% | 99.0% |
| DV1/COLOMBIA/514/2013 | 97.8% | 94.8% | 94.1% |
| DV1/COLOMBIA/515/2013 | 99.4% | 99.1% | 98.9% |
| DV1/COLOMBIA/516/2013 | 99.5% | 99.2% | 98.4% |
| DV1/COLOMBIA/517/2013 | 99.7% | 99.1% | 98.9% |
| DV1/COLOMBIA/518/2013 | 99.6% | 99.4% | 99.2% |
| DV1/COLOMBIA/519/2013 | 99.7% | 99.4% | 99.0% |
| DV1/COLOMBIA/520/2012 | 97.5% | 94.7% | 90.9% |
| DV1/COLOMBIA/521/2013 | 92.0% | 83.6% | 80.6% |
| DV1/COLOMBIA/522/2013 | 99.4% | 98.5% | 97.7% |
| DV1/COLOMBIA/523/2013 | 98.5% | 94.6% | 89.5% |
| DV1/COLOMBIA/524/2013 | 99.7% | 99.3% | 98.6% |
| DV1/COLOMBIA/525/2011 | 94.4% | 88.7% | 83.6% |
| DV1/COLOMBIA/526/2013 | 99.4% | 99.0% | 98.7% |
| DV1/COLOMBIA/527/2012 | 99.1% | 98.7% | 98.5% |
| DV1/COLOMBIA/528/2013 | 99.0% | 97.6% | 95.5% |
| DV1/HONDURAS/139/2013 | 99.8% | 99.6% | 99.0% |
| DV1/HONDURAS/352/2014 | 99.6% | 99.1% | 98.8% |
| DV1/HONDURAS/361/2013 | 99.9% | 99.7% | 99.4% |
| DV1/INDONESIA/364/2013 | 99.9% | 99.6% | 99.1% |
| DV1/INDONESIA/365/2013 | 99.8% | 99.6% | 98.9% |
| DV1/INDONESIA/366/2013 | 99.8% | 99.6% | 99.0% |
| DV1/INDONESIA/384/2013 | 99.6% | 99.4% | 99.0% |
| DV1/INDONESIA/385/2013 | 99.9% | 99.6% | 99.1% |
| DV1/INDONESIA/386/2013 | 99.3% | 98.0% | 96.2% |
| DV1/INDONESIA/387/2013 | 100.0% | 99.6% | 99.3% |
| DV1/INDONESIA/388/2013 | 99.7% | 99.5% | 99.1% |
| DV1/INDONESIA/389/2013 | 99.5% | 99.2% | 98.8% |
| DV1/INDONESIA/390/2013 | 99.8% | 99.4% | 98.8% |
| DV1/INDONESIA/391/2013 | 99.8% | 99.4% | 99.1% |
| DV1/INDONESIA/392/2013 | 99.4% | 99.1% | 98.8% |
| DV1/MALAYSIA/168/2012 | 99.6% | 99.2% | 98.3% |
| DV1/MALAYSIA/187/2011 | 97.8% | 95.2% | 89.8% |
| DV1/MEXICO/140/2012 | 99.8% | 99.8% | 99.6% |
| DV1/MEXICO/141/2012 | 99.9% | 99.7% | 99.6% |
| DV1/MEXICO/142/2012 | 99.8% | 99.4% | 99.2% |
| DV1/MEXICO/143/2012 | 99.8% | 99.7% | 99.6% |
| DV1/MEXICO/144/2012 | 99.9% | 99.7% | 99.5% |
| DV1/MEXICO/354/2013 | 99.5% | 99.3% | 99.0% |
| DV1/MEXICO/355/2013 | 99.9% | 99.7% | 98.9% |
| DV1/MEXICO/357/2013 | 99.5% | 99.2% | 99.0% |
| DV1/MEXICO/359/2013 | 99.4% | 99.1% | 97.8% |
| DV1/MEXICO/362/2013 | 100.0% | 99.7% | 98.9% |
| DV1/MEXICO/529/2012 | 99.6% | 99.4% | 98.8% |
| DV1/MEXICO/530/2013 | 99.6% | 99.2% | 98.9% |
| DV1/MEXICO/531/2012 | 99.9% | 99.6% | 99.1% |
| DV1/MEXICO/532/2012 | 99.5% | 99.2% | 98.8% |
| DV1/MEXICO/533/2012 | 99.9% | 99.6% | 99.3% |
| DV1/MEXICO/534/2012 | 99.5% | 98.8% | 98.2% |
| DV1/MEXICO/535/2012 | 97.7% | 96.0% | 95.6% |
| DV1/MEXICO/536/2012 | 99.7% | 99.3% | 99.0% |
| DV1/MEXICO/537/2012 | 99.5% | 99.2% | 98.6% |
| DV1/MEXICO/538/2012 | 97.4% | 96.8% | 95.5% |
| DV1/MEXICO/539/2012 | 99.8% | 99.6% | 99.2% |
| DV1/MEXICO/540/2012 | 98.9% | 98.4% | 97.6% |
| DV1/MEXICO/541/2012 | 99.7% | 99.4% | 98.6% |
| DV1/MEXICO/542/2012 | 96.9% | 95.8% | 94.5% |
| DV1/MEXICO/543/2012 | 95.1% | 93.8% | 93.3% |
| DV1/MEXICO/544/2012 | 99.5% | 98.8% | 97.2% |
| DV1/MEXICO/545/2012 | 99.8% | 99.4% | 98.9% |
| DV1/MEXICO/546/2012 | 99.5% | 99.1% | 97.9% |
| DV1/MEXICO/547/2012 | 99.2% | 98.8% | 97.6% |
| DV1/MEXICO/548/2012 | 99.5% | 98.9% | 97.7% |
| DV1/MEXICO/549/2012 | 96.2% | 93.0% | 91.2% |
| DV1/MEXICO/550/2012 | 99.5% | 99.2% | 98.6% |
| DV1/PHILIPPINES/001/2011 | 99.9% | 99.6% | 99.4% |
| DV1/PHILIPPINES/002/2011 | 99.9% | 99.8% | 99.5% |
| DV1/PHILIPPINES/003/2011 | 100.0% | 99.9% | 99.6% |
| DV1/PHILIPPINES/004/2011 | 100.0% | 99.8% | 99.7% |
| DV1/PHILIPPINES/005/2011 | 99.9% | 99.7% | 99.6% |
| DV1/PHILIPPINES/006/2012 | 99.9% | 99.6% | 99.1% |
| DV1/PHILIPPINES/007/2012 | 100.0% | 99.6% | 99.3% |
| DV1/PHILIPPINES/008/2012 | 99.5% | 99.0% | 98.8% |
| DV1/PHILIPPINES/009/2012 | 100.0% | 99.9% | 99.7% |
| DV1/PHILIPPINES/010/2012 | 100.0% | 99.5% | 99.2% |
| DV1/PHILIPPINES/011/2012 | 97.4% | 89.3% | 82.5% |
| DV1/PHILIPPINES/012/2012 | 100.0% | 99.8% | 99.5% |
| DV1/PHILIPPINES/013/2012 | 99.9% | 99.6% | 99.6% |
| DV1/PHILIPPINES/014/2012 | 100.0% | 99.7% | 99.5% |
| DV1/PHILIPPINES/015/2012 | 99.9% | 99.8% | 99.6% |
| DV1/PHILIPPINES/159/2012 | 99.5% | 99.0% | 98.6% |
| DV1/PHILIPPINES/160/2012 | 99.3% | 99.0% | 98.7% |
| DV1/PHILIPPINES/161/2012 | 99.4% | 98.9% | 98.7% |
| DV1/PHILIPPINES/162/2012 | 99.7% | 99.4% | 99.1% |
| DV1/PHILIPPINES/163/2012 | 99.7% | 99.3% | 98.7% |
| DV1/PHILIPPINES/164/2012 | 99.7% | 99.2% | 99.0% |
| DV1/PHILIPPINES/165/2012 | 99.6% | 99.3% | 99.0% |
| DV1/PHILIPPINES/166/2011 | 99.2% | 98.7% | 97.0% |
| DV1/PHILIPPINES/167/2012 | 99.1% | 98.8% | 98.4% |
| DV1/PHILIPPINES/183/2011 | 99.7% | 99.4% | 98.4% |
| DV1/PHILIPPINES/184/2011 | 99.6% | 99.1% | 98.7% |
| DV1/PHILIPPINES/185/2011 | 99.7% | 99.1% | 98.0% |
| DV1/PHILIPPINES/186/2012 | 99.4% | 98.9% | 97.9% |
| DV1/PHILIPPINES/188/2011 | 99.5% | 99.0% | 98.7% |
| DV1/PHILIPPINES/367/2013 | 99.8% | 99.6% | 99.0% |
| DV1/PHILIPPINES/368/2013 | 99.8% | 99.5% | 99.1% |
| DV1/PHILIPPINES/369/2012 | 99.6% | 99.3% | 97.8% |
| DV1/PHILIPPINES/370/2013 | 99.7% | 99.2% | 97.7% |
| DV1/PHILIPPINES/371/2013 | 99.9% | 99.5% | 99.2% |
| DV1/PHILIPPINES/372/2013 | 99.4% | 97.9% | 97.0% |
| DV1/PHILIPPINES/373/2013 | 99.8% | 99.6% | 99.3% |
| DV1/PHILIPPINES/374/2012 | 99.7% | 99.5% | 99.2% |
| DV1/PHILIPPINES/375/2012 | 99.6% | 99.2% | 98.8% |
| DV1/PHILIPPINES/376/2012 | 98.8% | 98.6% | 98.3% |
| DV1/PHILIPPINES/377/2012 | 100.0% | 99.6% | 99.4% |
| DV1/PHILIPPINES/378/2013 | 99.0% | 98.5% | 97.9% |
| DV1/PHILIPPINES/379/2012 | 99.5% | 99.2% | 98.2% |
| DV1/PHILIPPINES/380/2012 | 99.8% | 99.6% | 99.6% |
| DV1/PHILIPPINES/381/2013 | 99.5% | 99.1% | 98.7% |
| DV1/PHILIPPINES/393/2013 | 99.4% | 99.1% | 98.9% |
| DV1/PHILIPPINES/394/2012 | 99.9% | 99.7% | 99.4% |
| DV1/PHILIPPINES/395/2012 | 99.4% | 97.3% | 95.3% |
| DV1/PHILIPPINES/396/2013 | 99.7% | 99.3% | 99.0% |
| DV1/PHILIPPINES/397/2013 | 99.8% | 99.6% | 99.4% |
| DV1/PHILIPPINES/398/2012 | 99.6% | 99.4% | 99.0% |
| DV1/PHILIPPINES/399/2012 | 99.5% | 99.3% | 99.0% |
| DV1/PHILIPPINES/400/2012 | 99.8% | 99.2% | 99.0% |
| DV1/PHILIPPINES/401/2012 | 99.6% | 99.2% | 98.9% |
| DV1/PHILIPPINES/402/2013 | 98.9% | 95.6% | 90.6% |
| DV1/PHILIPPINES/403/2013 | 99.7% | 99.6% | 99.3% |
| DV1/PHILIPPINES/404/2012 | 99.9% | 99.8% | 99.0% |
| DV1/PHILIPPINES/405/2013 | 99.6% | 99.4% | 99.1% |
| DV1/PHILIPPINES/406/2013 | 99.7% | 99.5% | 99.0% |
| DV1/PHILIPPINES/407/2013 | 99.2% | 98.7% | 98.1% |
| DV1/PHILIPPINES/408/2012 | 99.9% | 99.8% | 99.5% |
| DV1/PHILIPPINES/409/2013 | 99.8% | 99.5% | 99.2% |
| DV1/PHILIPPINES/410/2013 | 99.3% | 97.4% | 97.0% |
| DV1/PHILIPPINES/411/2013 | 99.5% | 99.2% | 98.7% |
| DV1/PHILIPPINES/412/2013 | 99.4% | 99.0% | 98.5% |
| DV1/PHILIPPINES/413/2012 | 99.8% | 99.5% | 99.2% |
| DV1/PHILIPPINES/414/2013 | 99.4% | 99.2% | 98.9% |
| DV1/PHILIPPINES/415/2012 | 99.2% | 99.0% | 98.7% |
| DV1/PHILIPPINES/416/2012 | 99.5% | 99.3% | 99.2% |
| DV1/PHILIPPINES/417/2012 | 99.5% | 99.1% | 98.9% |
| DV1/PHILIPPINES/418/2013 | 99.7% | 99.2% | 98.9% |
| DV1/PHILIPPINES/419/2012 | 99.4% | 99.3% | 99.0% |
| DV1/PHILIPPINES/420/2012 | 99.6% | 99.1% | 98.8% |
| DV1/PHILIPPINES/421/2013 | 99.8% | 99.6% | 99.5% |
| DV1/PHILIPPINES/422/2013 | 99.8% | 99.5% | 99.2% |
| DV1/PHILIPPINES/423/2013 | 99.7% | 99.4% | 99.1% |
| DV1/PHILIPPINES/424/2012 | 99.8% | 99.6% | 99.4% |
| DV1/PHILIPPINES/429/2011 | 99.7% | 99.4% | 99.2% |
| DV1/PHILIPPINES/431/2012 | 99.3% | 97.3% | 95.3% |
| DV1/PHILIPPINES/432/2012 | 99.1% | 96.8% | 94.4% |
| DV1/PHILIPPINES/433/2013 | 98.0% | 97.7% | 97.3% |
| DV1/PHILIPPINES/434/2013 | 99.7% | 99.4% | 99.3% |
| DV1/PHILIPPINES/435/2013 | 99.3% | 97.1% | 93.8% |
| DV1/PHILIPPINES/437/2013 | 99.8% | 99.4% | 99.2% |
| DV1/PHILIPPINES/438/2012 | 99.4% | 99.0% | 98.9% |
| DV1/PHILIPPINES/439/2012 | 98.9% | 97.0% | 94.6% |
| DV1/PHILIPPINES/440/2012 | 99.7% | 98.7% | 97.5% |
| DV1/PHILIPPINES/659/2013 | 99.1% | 98.2% | 96.9% |
| DV1/PHILIPPINES/660/2012 | 96.6% | 91.9% | 85.2% |
| DV1/PUERTORICO/330/2013 | #N/A | #N/A | #N/A |
| DV1/PUERTORICO/331/2012 | #N/A | #N/A | #N/A |
| DV1/PUERTORICO/334/2012 | #N/A | #N/A | #N/A |
| DV1/PUERTORICO/335/2012 | #N/A | #N/A | #N/A |
| DV1/PUERTORICO/363/2013 | #N/A | #N/A | #N/A |
| DV1/PUERTORICO/551/2013 | 99.7% | 99.4% | 99.3% |
| DV1/PUERTORICO/552/2013 | 99.7% | 99.3% | 98.9% |
| DV1/PUERTORICO/553/2013 | 99.8% | 99.6% | 99.1% |
| DV1/PUERTORICO/554/2012 | 98.3% | 97.5% | 97.0% |
| DV1/PUERTORICO/555/2013 | 99.8% | 99.5% | 99.0% |
| DV1/PUERTORICO/556/2013 | 99.7% | 99.4% | 98.9% |
| DV1/PUERTORICO/557/2013 | 99.8% | 99.6% | 99.3% |
| DV1/PUERTORICO/558/2013 | 97.6% | 95.5% | 94.5% |
| DV1/PUERTORICO/559/2013 | 99.9% | 99.4% | 98.6% |
| DV1/THAILAND/169/2012 | 99.9% | 99.7% | 99.3% |
| DV1/THAILAND/170/2012 | 99.9% | 99.6% | 99.1% |
| DV1/THAILAND/171/2012 | 99.6% | 99.1% | 98.6% |
| DV1/THAILAND/172/2012 | 95.7% | 89.5% | 86.3% |
| DV1/THAILAND/173/2012 | 99.5% | 98.9% | 98.5% |
| DV1/THAILAND/174/2012 | 99.8% | 99.4% | 98.9% |
| DV1/THAILAND/175/2012 | 99.6% | 99.3% | 98.6% |
| DV1/THAILAND/176/2012 | 99.9% | 99.6% | 99.3% |
| DV1/THAILAND/382/2013 | 99.7% | 99.2% | 99.0% |
| DV1/THAILAND/383/2013 | 99.9% | 99.8% | 99.3% |
| DV1/THAILAND/425/2013 | 99.3% | 98.9% | 98.3% |
| DV1/THAILAND/426/2013 | 91.8% | 79.7% | 70.7% |
| DV1/THAILAND/427/2013 | 98.4% | 96.6% | 95.7% |
| DV1/THAILAND/428/2013 | 99.9% | 99.4% | 99.4% |
| DV1/THAILAND/430/2013 | 99.9% | 99.7% | 99.6% |
| DV1/VIETNAM/177/2012 | 99.5% | 99.2% | 98.7% |
| DV1/VIETNAM/178/2011 | 99.6% | 99.5% | 99.2% |
| DV1/VIETNAM/179/2011 | 90.0% | 81.7% | 76.9% |
| DV1/VIETNAM/180/2012 | 95.4% | 89.6% | 87.2% |
| DV1/VIETNAM/181/2012 | 98.8% | 96.9% | 95.7% |
| DV1/VIETNAM/182/2012 | 99.8% | 99.5% | 99.1% |
| DV1/VIETNAM/436/2013 | 98.9% | 98.5% | 97.5% |
| DV2/BRAZIL/229/2013 | 99.8% | 99.6% | 99.3% |
| DV2/COLOMBIA/234/2012 | 100.0% | 99.9% | 99.7% |
| DV2/COLOMBIA/235/2013 | 99.9% | 99.4% | 98.2% |
| DV2/COLOMBIA/236/2013 | 99.8% | 99.7% | 99.4% |
| DV2/COLOMBIA/237/2012 | 99.9% | 99.9% | 99.7% |
| DV2/COLOMBIA/238/2012 | 99.9% | 99.7% | 99.4% |
| DV2/COLOMBIA/239/2012 | 99.9% | 99.8% | 99.5% |
| DV2/COLOMBIA/240/2012 | 99.9% | 99.7% | 99.4% |
| DV2/COLOMBIA/241/2012 | 100.0% | 99.8% | 99.5% |
| DV2/COLOMBIA/242/2012 | 99.8% | 99.6% | 99.5% |
| DV2/COLOMBIA/243/2012 | 99.9% | 99.8% | 99.5% |
| DV2/COLOMBIA/244/2012 | 99.8% | 99.4% | 99.4% |
| DV2/COLOMBIA/245/2012 | 100.0% | 99.8% | 99.7% |
| DV2/COLOMBIA/246/2013 | 99.8% | 99.5% | 99.1% |
| DV2/COLOMBIA/247/2013 | 99.9% | 99.9% | 99.7% |
| DV2/COLOMBIA/248/2012 | 99.8% | 99.7% | 99.6% |
| DV2/COLOMBIA/249/2013 | 99.8% | 99.3% | 98.9% |
| DV2/COLOMBIA/250/2013 | 100.0% | 99.6% | 99.2% |
| DV2/COLOMBIA/251/2013 | 99.7% | 99.3% | 99.1% |
| DV2/COLOMBIA/252/2013 | 99.9% | 99.4% | 98.6% |
| DV2/COLOMBIA/253/2013 | 99.6% | 99.3% | 98.9% |
| DV2/COLOMBIA/254/2013 | 99.5% | 99.4% | 99.2% |
| DV2/COLOMBIA/255/2013 | 99.8% | 99.7% | 99.6% |
| DV2/COLOMBIA/256/2013 | 99.9% | 99.5% | 98.7% |
| DV2/COLOMBIA/257/2013 | 99.5% | 98.7% | 97.8% |
| DV2/COLOMBIA/258/2012 | 99.5% | 99.4% | 99.3% |
| DV2/COLOMBIA/259/2012 | 99.7% | 99.1% | 98.6% |
| DV2/COLOMBIA/260/2013 | 99.6% | 98.3% | 95.1% |
| DV2/COLOMBIA/261/2012 | 98.9% | 98.0% | 96.8% |
| DV2/COLOMBIA/273/2011 | 99.5% | 99.1% | 98.4% |
| DV2/COLOMBIA/274/2012 | 99.7% | 98.4% | 96.1% |
| DV2/COLOMBIA/275/2012 | 98.0% | 96.8% | 96.0% |
| DV2/COLOMBIA/276/2014 | 94.8% | 90.2% | 84.3% |
| DV2/COLOMBIA/277/2013 | 98.1% | 96.3% | 95.9% |
| DV2/COLOMBIA/278/2013 | 99.5% | 98.6% | 98.2% |
| DV2/COLOMBIA/279/2013 | 99.2% | 98.2% | 96.9% |
| DV2/HONDURAS/189/2013 | 99.1% | 98.6% | 98.1% |
| DV2/HONDURAS/190/2013 | 99.5% | 95.8% | 93.0% |
| DV2/HONDURAS/191/2013 | 99.8% | 99.7% | 99.3% |
| DV2/HONDURAS/192/2013 | 99.4% | 98.9% | 98.6% |
| DV2/HONDURAS/193/2012 | 99.4% | 99.0% | 98.8% |
| DV2/HONDURAS/194/2013 | 100.0% | 99.8% | 99.7% |
| DV2/HONDURAS/195/2012 | 99.3% | 98.9% | 98.5% |
| DV2/HONDURAS/196/2013 | 99.8% | 99.7% | 99.3% |
| DV2/HONDURAS/224/2012 | 99.1% | 98.6% | 98.1% |
| DV2/HONDURAS/225/2013 | 99.4% | 99.1% | 98.7% |
| DV2/HONDURAS/230/2013 | 98.7% | 96.8% | 96.5% |
| DV2/HONDURAS/231/2013 | 99.8% | 99.4% | 98.9% |
| DV2/HONDURAS/232/2013 | 99.8% | 99.7% | 99.3% |
| DV2/HONDURAS/233/2013 | 99.9% | 99.7% | 99.4% |
| DV2/HONDURAS/280/2013 | 99.6% | 99.2% | 98.6% |
| DV2/HONDURAS/281/2013 | 99.4% | 98.4% | 97.8% |
| DV2/HONDURAS/282/2013 | 97.9% | 93.3% | 91.9% |
| DV2/HONDURAS/283/2013 | 88.4% | 80.8% | 76.9% |
| DV2/HONDURAS/284/2013 | 99.6% | 99.0% | 97.6% |
| DV2/HONDURAS/285/2013 | 99.5% | 98.8% | 98.2% |
| DV2/HONDURAS/286/2013 | 99.4% | 99.0% | 98.6% |
| DV2/INDONESIA/016/2013 | 98.5% | 96.9% | 95.5% |
| DV2/INDONESIA/018/2013 | 99.8% | 99.3% | 98.6% |
| DV2/INDONESIA/019/2012 | 99.4% | 98.3% | 97.2% |
| DV2/INDONESIA/023/2013 | 93.3% | 92.2% | 89.9% |
| DV2/INDONESIA/024/2012 | 98.9% | 97.8% | 96.5% |
| DV2/INDONESIA/025/2012 | 91.1% | 87.3% | 85.8% |
| DV2/INDONESIA/040/2013 | 99.9% | 99.6% | 99.4% |
| DV2/INDONESIA/041/2012 | 99.8% | 99.6% | 99.4% |
| DV2/INDONESIA/042/2012 | 98.4% | 96.6% | 95.7% |
| DV2/MALAYSIA/039/2013 | 99.7% | 99.5% | 99.3% |
| DV2/MALAYSIA/311/2011 | 99.6% | 99.4% | 98.9% |
| DV2/MEXICO/197/2012 | 100.0% | 99.8% | 99.5% |
| DV2/MEXICO/198/2012 | 99.4% | 99.2% | 98.7% |
| DV2/MEXICO/199/2012 | 99.7% | 99.5% | 98.7% |
| DV2/MEXICO/200/2013 | 99.9% | 99.7% | 99.4% |
| DV2/MEXICO/201/2013 | 99.8% | 99.6% | 99.3% |
| DV2/MEXICO/202/2013 | 99.9% | 99.6% | 99.5% |
| DV2/MEXICO/203/2012 | 99.9% | 99.7% | 99.6% |
| DV2/MEXICO/204/2013 | 99.9% | 99.6% | 98.7% |
| DV2/MEXICO/205/2012 | 99.6% | 99.5% | 99.4% |
| DV2/MEXICO/206/2013 | 99.7% | 99.2% | 99.1% |
| DV2/MEXICO/207/2013 | 99.6% | 99.5% | 99.2% |
| DV2/MEXICO/208/2013 | 99.8% | 99.6% | 99.4% |
| DV2/MEXICO/209/2012 | 99.4% | 98.3% | 97.6% |
| DV2/MEXICO/210/2012 | 100.0% | 99.9% | 99.7% |
| DV2/MEXICO/211/2013 | 99.9% | 99.6% | 99.3% |
| DV2/MEXICO/212/2013 | 99.8% | 99.7% | 99.2% |
| DV2/MEXICO/213/2012 | 98.6% | 97.7% | 95.9% |
| DV2/MEXICO/214/2012 | 100.0% | 99.9% | 99.2% |
| DV2/MEXICO/215/2013 | 99.8% | 99.4% | 98.7% |
| DV2/MEXICO/216/2013 | 100.0% | 99.8% | 99.7% |
| DV2/MEXICO/217/2013 | 100.0% | 99.7% | 99.6% |
| DV2/MEXICO/218/2013 | 99.9% | 99.6% | 99.4% |
| DV2/MEXICO/219/2013 | 99.8% | 99.5% | 99.3% |
| DV2/MEXICO/220/2013 | 99.9% | 99.5% | 99.2% |
| DV2/MEXICO/221/2011 | 99.8% | 99.6% | 99.1% |
| DV2/MEXICO/222/2012 | 99.9% | 99.7% | 99.7% |
| DV2/MEXICO/223/2011 | 99.7% | 99.5% | 98.5% |
| DV2/MEXICO/226/2012 | 99.7% | 99.2% | 98.7% |
| DV2/MEXICO/227/2012 | 99.5% | 99.2% | 97.9% |
| DV2/MEXICO/228/2013 | 98.6% | 95.3% | 91.1% |
| DV2/MEXICO/262/2013 | 99.9% | 99.7% | 99.4% |
| DV2/MEXICO/263/2012 | 99.8% | 99.5% | 99.4% |
| DV2/MEXICO/264/2011 | 99.9% | 99.8% | 99.7% |
| DV2/MEXICO/265/2013 | 99.7% | 99.5% | 99.2% |
| DV2/MEXICO/266/2012 | 100.0% | 99.9% | 99.8% |
| DV2/MEXICO/267/2013 | 100.0% | 99.8% | 99.7% |
| DV2/MEXICO/268/2012 | 99.9% | 99.7% | 99.4% |
| DV2/MEXICO/269/2013 | 99.9% | 99.7% | 99.5% |
| DV2/MEXICO/270/2012 | 99.2% | 96.4% | 92.5% |
| DV2/MEXICO/271/2013 | 99.8% | 99.6% | 99.3% |
| DV2/MEXICO/272/2012 | 99.8% | 99.5% | 99.1% |
| DV2/MEXICO/661/2012 | 97.5% | 95.6% | 93.6% |
| DV2/PHILIPPINES/017/2013 | 92.9% | 84.6% | 77.5% |
| DV2/PHILIPPINES/020/2013 | 95.7% | 86.9% | 83.3% |
| DV2/PHILIPPINES/021/2013 | 98.8% | 97.4% | 95.3% |
| DV2/PHILIPPINES/026/2013 | 98.0% | 97.3% | 96.1% |
| DV2/PHILIPPINES/027/2013 | 93.9% | 88.8% | 84.0% |
| DV2/PHILIPPINES/028/2013 | 99.0% | 97.9% | 97.2% |
| DV2/PHILIPPINES/029/2013 | 98.4% | 97.5% | 96.5% |
| DV2/PHILIPPINES/030/2013 | 99.1% | 97.8% | 96.3% |
| DV2/PHILIPPINES/031/2013 | 87.8% | 84.9% | 81.1% |
| DV2/PHILIPPINES/032/2013 | 93.0% | 90.1% | 89.0% |
| DV2/PHILIPPINES/033/2013 | 98.8% | 97.1% | 94.5% |
| DV2/PHILIPPINES/043/2013 | 99.9% | 99.6% | 99.3% |
| DV2/PHILIPPINES/044/2013 | 99.9% | 99.7% | 99.6% |
| DV2/PHILIPPINES/045/2013 | 99.9% | 99.4% | 99.4% |
| DV2/PHILIPPINES/046/2012 | 99.9% | 99.6% | 99.4% |
| DV2/PHILIPPINES/047/2013 | 99.7% | 99.4% | 99.1% |
| DV2/PHILIPPINES/048/2013 | 99.9% | 99.0% | 97.9% |
| DV2/PHILIPPINES/049/2013 | 100.0% | 99.9% | 99.8% |
| DV2/PHILIPPINES/050/2013 | 99.9% | 99.8% | 99.7% |
| DV2/PHILIPPINES/051/2013 | 99.8% | 99.6% | 99.5% |
| DV2/PHILIPPINES/052/2013 | 99.8% | 99.5% | 99.2% |
| DV2/PHILIPPINES/053/2013 | 99.7% | 99.4% | 99.2% |
| DV2/PHILIPPINES/054/2013 | 93.7% | 92.3% | 91.8% |
| DV2/PHILIPPINES/055/2013 | 99.8% | 99.4% | 99.3% |
| DV2/PHILIPPINES/056/2013 | 99.8% | 99.7% | 99.4% |
| DV2/PHILIPPINES/057/2013 | 99.6% | 99.2% | 98.4% |
| DV2/PHILIPPINES/058/2013 | 99.1% | 97.3% | 96.7% |
| DV2/PHILIPPINES/287/2012 | 99.7% | 99.7% | 99.4% |
| DV2/PHILIPPINES/288/2012 | 100.0% | 99.9% | 99.8% |
| DV2/PHILIPPINES/289/2012 | 99.8% | 99.5% | 99.0% |
| DV2/PHILIPPINES/290/2012 | 99.7% | 99.5% | 99.3% |
| DV2/PHILIPPINES/291/2012 | 99.9% | 99.9% | 99.9% |
| DV2/PHILIPPINES/292/2012 | 100.0% | 99.7% | 99.5% |
| DV2/PHILIPPINES/293/2012 | 100.0% | 99.8% | 98.9% |
| DV2/PHILIPPINES/294/2012 | 99.8% | 99.7% | 98.0% |
| DV2/PHILIPPINES/295/2012 | 99.9% | 99.8% | 99.2% |
| DV2/PHILIPPINES/296/2012 | 99.8% | 99.6% | 99.2% |
| DV2/PHILIPPINES/312/2013 | 99.6% | 99.1% | 98.5% |
| DV2/THAILAND/034/2013 | 89.8% | 82.6% | 79.6% |
| DV2/THAILAND/035/2012 | 94.5% | 91.1% | 89.5% |
| DV2/THAILAND/037/2012 | 91.1% | 86.6% | 85.1% |
| DV2/THAILAND/038/2013 | 91.3% | 85.5% | 82.2% |
| DV2/THAILAND/059/2013 | 99.6% | 99.2% | 98.9% |
| DV2/THAILAND/060/2012 | 99.8% | 99.6% | 99.3% |
| DV2/THAILAND/061/2013 | 86.1% | 75.1% | 69.5% |
| DV2/THAILAND/063/2013 | 91.8% | 83.7% | 76.4% |
| DV2/THAILAND/313/2012 | 99.6% | 99.1% | 98.7% |
| DV2/THAILAND/314/2012 | 98.2% | 94.1% | 90.5% |
| DV2/THAILAND/315/2012 | 99.4% | 98.9% | 98.5% |
| DV2/THAILAND/316/2011 | 99.1% | 97.9% | 96.6% |
| DV2/THAILAND/317/2012 | 99.6% | 99.2% | 98.5% |
| DV2/THAILAND/318/2012 | 99.7% | 98.8% | 98.1% |
| DV2/THAILAND/319/2012 | 99.1% | 97.6% | 96.1% |
| DV2/THAILAND/320/2012 | 99.6% | 99.2% | 98.7% |
| DV2/THAILAND/321/2012 | 99.5% | 99.1% | 98.5% |
| DV2/THAILAND/322/2012 | 99.4% | 98.8% | 98.3% |
| DV2/THAILAND/323/2012 | 98.9% | 98.0% | 97.5% |
| DV2/THAILAND/324/2012 | 99.7% | 99.2% | 98.7% |
| DV2/THAILAND/325/2012 | 98.6% | 96.2% | 94.3% |
| DV2/THAILAND/326/2012 | 95.9% | 93.3% | 92.0% |
| DV2/THAILAND/327/2012 | 99.6% | 99.2% | 98.9% |
| DV2/THAILAND/328/2012 | 99.0% | 97.9% | 96.2% |
| DV2/THAILAND/329/2012 | 98.9% | 98.3% | 97.1% |
| DV2/VIETNAM/022/2012 | 96.7% | 94.9% | 93.3% |
| DV2/VIETNAM/036/2013 | 81.8% | 65.6% | 61.0% |
| DV2/VIETNAM/062/2012 | 99.8% | 99.6% | 99.1% |
| DV2/VIETNAM/297/2012 | 99.4% | 98.6% | 98.2% |
| DV2/VIETNAM/298/2011 | 98.3% | 97.0% | 96.4% |
| DV2/VIETNAM/299/2012 | 99.0% | 98.1% | 96.5% |
| DV2/VIETNAM/300/2012 | 99.4% | 99.0% | 98.4% |
| DV2/VIETNAM/301/2012 | 89.4% | 81.4% | 75.3% |
| DV2/VIETNAM/302/2012 | 98.6% | 97.9% | 97.1% |
| DV2/VIETNAM/303/2012 | 99.4% | 98.9% | 98.3% |
| DV2/VIETNAM/304/2012 | 99.4% | 99.0% | 98.5% |
| DV2/VIETNAM/305/2012 | 98.9% | 97.7% | 94.0% |
| DV2/VIETNAM/306/2012 | 95.3% | 94.8% | 94.4% |
| DV2/VIETNAM/307/2012 | 99.8% | 99.6% | 98.9% |
| DV2/VIETNAM/308/2011 | 99.7% | 99.0% | 98.4% |
| DV2/VIETNAM/309/2012 | 99.7% | 99.0% | 98.7% |
| DV2/VIETNAM/310/2012 | 99.5% | 99.2% | 98.6% |
| DV2/VIETNAM/662/2013 | 99.2% | 98.8% | 98.3% |
| DV3/COLOMBIA/448/2012 | 100.0% | 99.6% | 99.3% |
| DV3/COLOMBIA/449/2012 | 99.8% | 99.5% | 99.3% |
| DV3/COLOMBIA/450/2013 | 99.8% | 99.3% | 98.5% |
| DV3/COLOMBIA/451/2013 | 99.4% | 99.0% | 98.1% |
| DV3/COLOMBIA/452/2013 | 99.9% | 99.6% | 99.3% |
| DV3/COLOMBIA/453/2013 | 99.1% | 98.5% | 98.1% |
| DV3/COLOMBIA/454/2013 | 99.7% | 99.3% | 98.8% |
| DV3/COLOMBIA/456/2013 | 99.9% | 99.4% | 99.2% |
| DV3/COLOMBIA/457/2013 | 100.0% | 99.4% | 99.0% |
| DV3/COLOMBIA/458/2013 | 99.9% | 99.6% | 99.3% |
| DV3/COLOMBIA/597/2013 | 99.0% | 98.3% | 97.8% |
| DV3/COLOMBIA/598/2013 | 99.7% | 99.3% | 99.0% |
| DV3/COLOMBIA/599/2012 | 99.4% | 99.1% | 99.0% |
| DV3/COLOMBIA/600/2013 | 99.7% | 98.7% | 98.5% |
| DV3/COLOMBIA/601/2013 | 99.6% | 99.1% | 98.7% |
| DV3/COLOMBIA/602/2013 | 98.5% | 97.1% | 96.7% |
| DV3/COLOMBIA/603/2012 | 99.3% | 98.7% | 98.2% |
| DV3/COLOMBIA/604/2013 | 99.6% | 98.8% | 98.1% |
| DV3/COLOMBIA/605/2013 | 99.7% | 99.1% | 98.7% |
| DV3/COLOMBIA/606/2013 | 99.7% | 99.3% | 99.0% |
| DV3/COLOMBIA/607/2012 | 99.7% | 99.2% | 98.7% |
| DV3/COLOMBIA/608/2013 | 99.6% | 99.3% | 99.0% |
| DV3/COLOMBIA/609/2013 | 99.3% | 98.8% | 98.2% |
| DV3/COLOMBIA/610/2013 | 99.5% | 99.1% | 98.7% |
| DV3/COLOMBIA/611/2012 | 99.8% | 99.6% | 99.4% |
| DV3/COLOMBIA/612/2012 | 99.7% | 99.5% | 98.7% |
| DV3/COLOMBIA/613/2013 | 99.7% | 99.4% | 99.0% |
| DV3/COLOMBIA/614/2012 | 99.7% | 99.4% | 98.9% |
| DV3/COLOMBIA/615/2013 | 99.3% | 99.0% | 98.6% |
| DV3/COLOMBIA/616/2013 | 99.5% | 99.0% | 98.5% |
| DV3/COLOMBIA/617/2013 | 99.6% | 99.1% | 98.9% |
| DV3/COLOMBIA/618/2013 | 99.6% | 99.1% | 98.7% |
| DV3/COLOMBIA/619/2013 | 99.2% | 98.1% | 97.8% |
| DV3/COLOMBIA/620/2013 | 99.5% | 99.3% | 99.2% |
| DV3/COLOMBIA/621/2012 | 99.7% | 99.1% | 98.6% |
| DV3/COLOMBIA/622/2012 | 99.5% | 98.9% | 98.5% |
| DV3/COLOMBIA/623/2012 | 99.1% | 97.4% | 91.5% |
| DV3/COLOMBIA/624/2012 | 99.3% | 99.2% | 98.9% |
| DV3/COLOMBIA/625/2012 | 99.3% | 98.8% | 98.2% |
| DV3/COLOMBIA/626/2013 | 99.6% | 99.3% | 98.7% |
| DV3/COLOMBIA/627/2013 | 99.3% | 98.9% | 98.5% |
| DV3/COLOMBIA/628/2013 | 99.3% | 98.9% | 98.4% |
| DV3/COLOMBIA/629/2012 | 99.7% | 99.5% | 99.4% |
| DV3/COLOMBIA/630/2013 | 99.6% | 99.4% | 99.2% |
| DV3/COLOMBIA/631/2012 | 99.6% | 99.3% | 98.7% |
| DV3/COLOMBIA/632/2013 | 99.7% | 99.4% | 99.0% |
| DV3/COLOMBIA/644/2013 | 99.8% | 99.6% | 99.5% |
| DV3/COLOMBIA/645/2013 | 99.8% | 99.6% | 99.4% |
| DV3/COLOMBIA/650/2012 | 99.8% | 99.3% | 98.7% |
| DV3/COLOMBIA/651/2013 | 99.7% | 99.2% | 98.6% |
| DV3/HONDURAS/459/2013 | 99.7% | 99.0% | 98.5% |
| DV3/HONDURAS/460/2013 | 99.6% | 99.4% | 99.2% |
| DV3/HONDURAS/461/2013 | 99.6% | 99.3% | 99.0% |
| DV3/HONDURAS/462/2013 | 99.6% | 99.2% | 98.6% |
| DV3/HONDURAS/463/2013 | 99.6% | 99.2% | 98.9% |
| DV3/HONDURAS/633/2013 | 99.6% | 99.2% | 98.7% |
| DV3/HONDURAS/634/2013 | 99.7% | 99.4% | 98.9% |
| DV3/HONDURAS/635/2013 | 99.7% | 98.8% | 98.4% |
| DV3/HONDURAS/646/2013 | 99.4% | 99.1% | 98.9% |
| DV3/HONDURAS/647/2013 | 99.9% | 99.6% | 99.5% |
| DV3/HONDURAS/648/2013 | 99.6% | 99.4% | 99.1% |
| DV3/HONDURAS/649/2013 | 99.6% | 99.0% | 98.7% |
| DV3/HONDURAS/652/2013 | 99.7% | 99.3% | 98.8% |
| DV3/HONDURAS/653/2013 | 99.9% | 99.7% | 99.3% |
| DV3/HONDURAS/654/2013 | 99.4% | 99.3% | 98.9% |
| DV3/HONDURAS/655/2013 | 99.6% | 99.4% | 99.2% |
| DV3/HONDURAS/656/2013 | 99.9% | 99.9% | 99.8% |
| DV3/HONDURAS/657/2013 | 99.8% | 99.6% | 99.3% |
| DV3/HONDURAS/658/2013 | 99.7% | 99.6% | 99.3% |
| DV3/INDONESIA/080/2013 | 99.7% | 99.6% | 99.5% |
| DV3/INDONESIA/081/2013 | 100.0% | 99.8% | 99.8% |
| DV3/INDONESIA/082/2013 | 99.7% | 99.6% | 99.4% |
| DV3/INDONESIA/083/2013 | 99.9% | 99.7% | 99.5% |
| DV3/INDONESIA/084/2013 | 99.9% | 99.8% | 99.8% |
| DV3/PHILIPPINES/074/2012 | 95.5% | 89.6% | 82.7% |
| DV3/PHILIPPINES/075/2012 | 99.8% | 99.4% | 99.1% |
| DV3/PHILIPPINES/076/2012 | 97.3% | 95.7% | 94.8% |
| DV3/PHILIPPINES/077/2012 | 99.6% | 99.6% | 99.5% |
| DV3/PHILIPPINES/078/2012 | 100.0% | 99.8% | 99.3% |
| DV3/PHILIPPINES/079/2012 | 99.5% | 99.3% | 99.1% |
| DV3/PHILIPPINES/441/2011 | 99.6% | 99.3% | 99.1% |
| DV3/PHILIPPINES/442/2012 | 98.4% | 96.8% | 95.7% |
| DV3/PHILIPPINES/443/2012 | 99.7% | 99.6% | 99.2% |
| DV3/PHILIPPINES/444/2012 | 99.7% | 99.3% | 98.9% |
| DV3/PHILIPPINES/636/2012 | 97.9% | 92.8% | 87.6% |
| DV3/PHILIPPINES/637/2012 | 99.6% | 99.1% | 98.9% |
| DV3/PHILIPPINES/638/2011 | 99.8% | 99.5% | 99.3% |
| DV3/PHILIPPINES/639/2012 | 100.0% | 99.6% | 99.5% |
| DV3/PHILIPPINES/640/2012 | 99.8% | 99.5% | 99.2% |
| DV3/PHILIPPINES/641/2012 | 97.7% | 96.7% | 94.8% |
| DV3/PHILIPPINES/642/2012 | 100.0% | 99.9% | 99.6% |
| DV3/PHILIPPINES/643/2012 | 99.0% | 97.1% | 92.3% |
| DV3/THAILAND/064/2013 | 99.5% | 98.9% | 98.2% |
| DV3/THAILAND/065/2013 | 99.8% | 99.6% | 99.2% |
| DV3/THAILAND/066/2013 | 99.8% | 99.4% | 99.1% |
| DV3/THAILAND/067/2013 | 97.9% | 97.5% | 97.2% |
| DV3/THAILAND/068/2013 | 99.7% | 99.5% | 99.3% |
| DV3/THAILAND/069/2013 | 99.8% | 99.7% | 99.5% |
| DV3/THAILAND/070/2013 | 99.9% | 99.5% | 99.2% |
| DV3/THAILAND/071/2013 | 99.7% | 99.2% | 98.6% |
| DV3/THAILAND/072/2013 | 100.0% | 99.7% | 99.4% |
| DV3/THAILAND/073/2013 | 99.9% | 99.7% | 99.5% |
| DV3/THAILAND/445/2013 | 99.4% | 98.9% | 98.2% |
| DV3/VIETNAM/085/2013 | 99.8% | 99.4% | 99.3% |
| DV3/VIETNAM/446/2012 | 100.0% | 99.5% | 98.9% |
| DV3/VIETNAM/447/2012 | 99.9% | 99.6% | 99.2% |
| DV3/VIETNAM/663/2011 | 99.3% | 98.9% | 98.1% |
| DV4/BRAZIL/497/2012 | 98.0% | 95.4% | 93.6% |
| DV4/BRAZIL/498/2012 | 88.8% | 80.5% | 79.6% |
| DV4/BRAZIL/499/2012 | 96.6% | 95.0% | 92.4% |
| DV4/BRAZIL/500/2013 | 94.7% | 89.9% | 87.7% |
| DV4/BRAZIL/501/2013 | 89.1% | 81.2% | 77.9% |
| DV4/BRAZIL/560/2012 | 99.7% | 99.2% | 98.7% |
| DV4/BRAZIL/561/2012 | 99.6% | 99.4% | 99.1% |
| DV4/BRAZIL/562/2012 | 99.3% | 99.1% | 98.8% |
| DV4/BRAZIL/563/2013 | 99.8% | 99.5% | 99.0% |
| DV4/BRAZIL/564/2012 | 99.9% | 99.7% | 99.2% |
| DV4/BRAZIL/565/2013 | 99.5% | 99.2% | 98.9% |
| DV4/BRAZIL/566/2012 | 99.9% | 99.5% | 99.3% |
| DV4/BRAZIL/567/2012 | 100.0% | 99.6% | 99.4% |
| DV4/BRAZIL/568/2012 | 99.9% | 99.4% | 99.0% |
| DV4/BRAZIL/569/2012 | 99.9% | 99.4% | 99.0% |
| DV4/BRAZIL/570/2013 | 99.7% | 99.3% | 99.1% |
| DV4/BRAZIL/571/2013 | 99.6% | 99.5% | 99.1% |
| DV4/BRAZIL/573/2012 | 99.8% | 99.5% | 99.0% |
| DV4/BRAZIL/574/2013 | 99.8% | 99.4% | 99.2% |
| DV4/BRAZIL/575/2013 | 99.8% | 99.6% | 99.2% |
| DV4/BRAZIL/576/2013 | 99.6% | 99.3% | 99.1% |
| DV4/BRAZIL/577/2013 | 99.2% | 98.9% | 97.9% |
| DV4/BRAZIL/578/2013 | 99.9% | 99.5% | 99.2% |
| DV4/BRAZIL/579/2013 | 97.4% | 94.8% | 92.5% |
| DV4/BRAZIL/580/2013 | 99.8% | 99.4% | 99.3% |
| DV4/BRAZIL/581/2013 | 99.6% | 99.4% | 99.0% |
| DV4/BRAZIL/582/2013 | 99.4% | 99.2% | 98.4% |
| DV4/BRAZIL/583/2013 | 99.7% | 99.4% | 99.2% |
| DV4/BRAZIL/584/2012 | 99.9% | 99.6% | 99.2% |
| DV4/BRAZIL/585/2012 | 99.4% | 98.9% | 98.6% |
| DV4/BRAZIL/586/2012 | 98.7% | 98.1% | 97.1% |
| DV4/BRAZIL/587/2012 | 99.7% | 99.3% | 99.0% |
| DV4/BRAZIL/588/2012 | 98.8% | 98.5% | 97.9% |
| DV4/BRAZIL/589/2012 | 98.4% | 98.1% | 97.8% |
| DV4/BRAZIL/590/2012 | 99.4% | 98.8% | 98.2% |
| DV4/BRAZIL/591/2012 | 99.1% | 98.7% | 98.4% |
| DV4/BRAZIL/592/2013 | 99.9% | 99.5% | 99.3% |
| DV4/BRAZIL/593/2012 | 99.7% | 99.3% | 98.8% |
| DV4/COLOMBIA/594/2013 | 99.7% | 99.3% | 98.8% |
| DV4/COLOMBIA/595/2013 | 99.7% | 98.2% | 97.9% |
| DV4/COLOMBIA/596/2013 | 99.6% | 99.4% | 98.9% |
| DV4/INDONESIA/086/2013 | 99.3% | 98.5% | 98.1% |
| DV4/MALAYSIA/087/2012 | 99.9% | 99.4% | 99.1% |
| DV4/MALAYSIA/088/2012 | 99.5% | 98.8% | 98.1% |
| DV4/MALAYSIA/464/2012 | 95.1% | 91.3% | 88.2% |
| DV4/MALAYSIA/465/2012 | 97.0% | 96.0% | 94.6% |
| DV4/MALAYSIA/664/2011 | 92.9% | 87.8% | 86.1% |
| DV4/PHILIPPINES/089/2013 | 99.5% | 98.6% | 98.0% |
| DV4/PHILIPPINES/090/2013 | 99.8% | 99.5% | 99.1% |
| DV4/PHILIPPINES/091/2013 | 97.5% | 94.8% | 93.4% |
| DV4/PHILIPPINES/092/2013 | 99.4% | 99.1% | 98.6% |
| DV4/PHILIPPINES/093/2013 | 99.8% | 99.3% | 98.9% |
| DV4/PHILIPPINES/094/2012 | 99.4% | 98.5% | 96.3% |
| DV4/PHILIPPINES/095/2013 | 99.1% | 98.0% | 97.2% |
| DV4/PHILIPPINES/096/2013 | 99.6% | 99.3% | 98.7% |
| DV4/PHILIPPINES/097/2013 | 99.8% | 99.2% | 98.8% |
| DV4/PHILIPPINES/098/2012 | 99.3% | 98.8% | 98.1% |
| DV4/PHILIPPINES/099/2013 | 99.1% | 98.7% | 98.2% |
| DV4/PHILIPPINES/100/2013 | 99.5% | 98.7% | 98.1% |
| DV4/PHILIPPINES/101/2013 | 99.9% | 99.3% | 98.7% |
| DV4/PHILIPPINES/102/2013 | 99.8% | 99.6% | 99.1% |
| DV4/PHILIPPINES/103/2013 | 99.6% | 98.9% | 98.3% |
| DV4/PHILIPPINES/104/2013 | 99.9% | 99.6% | 99.1% |
| DV4/PHILIPPINES/105/2013 | 99.8% | 99.2% | 99.0% |
| DV4/PHILIPPINES/106/2013 | 99.9% | 99.6% | 99.3% |
| DV4/PHILIPPINES/107/2013 | 99.6% | 98.8% | 98.2% |
| DV4/PHILIPPINES/108/2013 | 99.8% | 97.2% | 88.1% |
| DV4/PHILIPPINES/109/2013 | 99.8% | 99.3% | 98.8% |
| DV4/PHILIPPINES/110/2012 | 99.5% | 99.2% | 98.7% |
| DV4/PHILIPPINES/111/2013 | 99.7% | 99.0% | 98.8% |
| DV4/PHILIPPINES/112/2013 | 99.7% | 99.5% | 99.2% |
| DV4/PHILIPPINES/113/2013 | 99.7% | 99.5% | 99.0% |
| DV4/PHILIPPINES/466/2012 | 96.7% | 94.3% | 92.5% |
| DV4/PHILIPPINES/467/2012 | 95.3% | 90.2% | 86.5% |
| DV4/PHILIPPINES/468/2012 | 93.7% | 88.1% | 87.5% |
| DV4/PHILIPPINES/469/2012 | 98.7% | 97.5% | 95.7% |
| DV4/PHILIPPINES/470/2012 | 97.6% | 96.1% | 93.8% |
| DV4/PUERTORICO/572/2012 | 99.6% | 99.3% | 98.9% |
| DV4/THAILAND/114/2013 | 99.9% | 99.6% | 99.3% |
| DV4/THAILAND/471/2012 | 99.4% | 98.6% | 96.8% |
| DV4/VIETNAM/115/2012 | 99.5% | 99.1% | 98.6% |
| DV4/VIETNAM/116/2013 | 99.6% | 99.5% | 99.0% |
| DV4/VIETNAM/117/2013 | 99.7% | 99.2% | 98.6% |
| DV4/VIETNAM/118/2013 | 99.5% | 99.2% | 98.6% |
| DV4/VIETNAM/119/2012 | 99.5% | 99.0% | 98.7% |
| DV4/VIETNAM/120/2013 | 99.7% | 99.6% | 99.2% |
| DV4/VIETNAM/121/2013 | 99.8% | 99.5% | 99.2% |
| DV4/VIETNAM/472/2012 | 99.0% | 97.3% | 94.6% |
| DV4/VIETNAM/473/2012 | 99.5% | 98.6% | 97.9% |
| DV4/VIETNAM/474/2012 | 99.2% | 98.0% | 96.7% |
| DV4/VIETNAM/475/2012 | 98.4% | 96.1% | 94.6% |
| DV4/VIETNAM/476/2012 | 98.9% | 97.9% | 97.0% |
| DV4/VIETNAM/477/2012 | 98.9% | 97.9% | 97.3% |
| DV4/VIETNAM/478/2012 | 99.5% | 98.9% | 98.4% |
| DV4/VIETNAM/479/2012 | 96.8% | 95.0% | 93.5% |
| DV4/VIETNAM/480/2012 | 99.7% | 99.1% | 98.0% |
| DV4/VIETNAM/481/2012 | 99.5% | 98.5% | 97.8% |
| DV4/VIETNAM/482/2012 | 99.3% | 98.7% | 98.2% |
| DV4/VIETNAM/483/2011 | 95.5% | 91.6% | 87.2% |
| DV4/VIETNAM/484/2012 | 96.2% | 94.2% | 91.6% |
| DV4/VIETNAM/485/2012 | 99.6% | 99.1% | 98.6% |
| DV4/VIETNAM/486/2012 | 98.7% | 97.7% | 96.2% |
| DV4/VIETNAM/487/2012 | 99.1% | 98.8% | 98.1% |
| DV4/VIETNAM/488/2012 | 98.4% | 96.8% | 95.4% |
| DV4/VIETNAM/489/2012 | 98.8% | 98.0% | 96.9% |
| DV4/VIETNAM/490/2011 | 99.4% | 99.1% | 98.4% |
| DV4/VIETNAM/491/2012 | 99.8% | 99.1% | 98.3% |
| DV4/VIETNAM/492/2012 | 97.2% | 90.2% | 85.2% |
| DV4/VIETNAM/493/2012 | 90.4% | 83.5% | 77.7% |
| DV4/VIETNAM/494/2012 | 98.1% | 96.4% | 93.9% |
| DV4/VIETNAM/495/2012 | 99.6% | 99.0% | 98.4% |
| DV4/VIETNAM/496/2012 | 99.2% | 98.7% | 97.8% |
